# Natural Gas Storage Filled with Peat-Derived Carbon Adsorbent: Influence of Nonisothermal Effects and Ethane Impurities on the Storage Cycle

**DOI:** 10.3390/nano12224066

**Published:** 2022-11-18

**Authors:** Andrey V. Shkolin, Evgeny M. Strizhenov, Sergey S. Chugaev, Ilya E. Men’shchikov, Viktoriia V. Gaidamavichute, Alexander E. Grinchenko, Anatoly A. Zherdev

**Affiliations:** 1 Research Institute of Power Engineering, Bauman Moscow State Technical University, Baumanskaya 2-ya str. 5, 105005 Moscow, Russia; 2 Frumkin Institute of Physical Chemistry and Electrochemistry, Russian Academy of Sciences, Leninskii Prospect, 31, Build. 4, 119071 Moscow, Russia

**Keywords:** mixture adsorption, nanoporous carbon, natural gas storage, methane, ethane, heat of adsorption, cyclic adsorption, ideal adsorbed solution theory, numerical molecular simulation, molecular dynamic

## Abstract

Adsorbed natural gas (ANG) is a promising solution for improving the safety and storage capacity of low-pressure gas storage systems. The structural–energetic and adsorption properties of active carbon ACPK, synthesized from cheap peat raw materials, are presented. Calculations of the methane–ethane mixture adsorption on ACPK were performed using the experimental adsorption isotherms of pure components. It is shown that the accumulation of ethane can significantly increase the energy capacity of the ANG storage. Numerical molecular modeling of the methane–ethane mixture adsorption in slit-like model micropores has been carried out. The molecular effects associated with the displacement of ethane by methane molecules and the formation of a molecule layered structure are shown. The integral molecular adsorption isotherm of the mixture according to the molecular modeling adequately corresponds to the ideal adsorbed solution theory (IAST). The cyclic processes of gas charging and discharging from the ANG storage based on the ACPK are simulated in three modes: adiabatic, isothermal, and thermocontrolled. The adiabatic mode leads to a loss of 27–33% of energy capacity at 3.5 MPa compared to the isothermal mode, which has a 9.4–19.5% lower energy capacity compared to the thermocontrolled mode, with more efficient desorption of both methane and ethane.

## 1. Introduction

Natural gas as one of the most common, cheap, and environmentally friendly fuels may in the near future become the main energy source for countries adhering to the principles of sustainable development. However, based on the experience of countries using natural gas as the main energy source for domestic consumption, it can be noted that among the main difficulties on the way to a large-scale energy transition to natural gas is the issue of gas delivery and back-up storage close to the consumer. The energy crisis in Europe in 2021–2022 has highlighted the importance of building an appropriate gas infrastructure with back-up storage to mitigate problems with gas supplies and uneven consumption. In addition, the events of 26 September 2022 on the Nord Stream gas pipelines also exposed the security problem of the existing gas infrastructure.

In recent years, the development of adsorbed natural gas (ANG) systems has been considered the most promising solution to improve the safety [1,2] and capacity of a low-pressure gas storage system [3]. However, there are several well-known difficulties that prevent ANG large-scale implementation: high requirements for the adsorbent, stability, uniformity and reproducibility of its adsorption properties; the exothermic nature of adsorption, leading to predominantly negative thermal effects, which should be compensated for during charging and discharging, and to an increase in the duration of these processes [4]; and a decrease in storage efficiency due to the accumulation of C_2+_ hydrocarbon impurities during cyclic operation [5].

The study of the impurities’ influence on the efficiency of adsorption gas storage is presented in the scientific literature much less often than studies on the thermal effects. This is mainly due to the complexity and laboriousness of conducting a cyclic experiment. A study [6] showed that when using natural gas with a methane content of about 92.18% mol., after 700 natural gas charging cycles, corresponding to about 250,000 km of vehicle mileage, the efficiency of the storage system drops by 50% due to the accumulation of impurity hydrocarbons C_2+_. The ethane accumulation ended around cycle 30, but the efficiency continued to decline through 700 cycles due to the accumulation of heavier hydrocarbons. In [6], an adsorption layer with high thermal conductivity due to expanded natural graphite (ENG) was used, which together with a water thermostat significantly reduced the thermal effects in the charging and discharging processes. The authors of [7] supplied gas in the radial direction from the central collector (pipes with 40 channels) to reduce the heterogeneity of thermal effects. The work used natural gas with a methane content of 90.68% mol. After 10 cycles (the study included 30 cycles in total), the charging mass of gas decreased by 20%, and the discharging mass of gas by only 2% compared with the first cycle: according to the authors, the loss in discharging due to the accumulation of impurities fully appeared already from the first cycle and subsequently weakly changed. In [8], the cyclic process of charging and discharging gas from an ANG tank at room temperature on commercial activated carbon Maxsorb MSC-30 was studied. When charging natural gas with 85.45% mol. methane, the gravimetric excess adsorption decreased to 33% after 100 cycles and continued to slowly decrease until it reached 25% by the 1000th cycle. Volumetric storage capacity decreased to 50% after the first 100 cycles and remained constant thereafter. The authors showed that periodic regeneration by degassing at 400 °C for 2 h makes it possible to remove impurities and reactivate active carbon. These works were not aimed to investigate the combined effect of the impurity accumulation and the thermal control of the adsorber or the thermal effects of adsorption. In [9], the method of mathematical modeling was used to study the cyclic processes of charging and discharging natural gas containing 88% mol. methane for two limit cases: isothermal and adiabatic. The authors noted that non-isothermal effects in the adiabatic process can be more negative to the performance of the gas charging–discharging cycle than the presence of impurities in natural gas. In the case of the adiabatic process using natural gas with impurities, the accumulation efficiency decreased by 37–40%. However, the authors noted that they limited themselves to only C_1_–C_4_ hydrocarbons, excluding the accumulation of heavier hydrocarbons from the model. The authors of [10] studied the effect of impurities in natural gas (methane content 90.58% mol.) in a full-size system with and without thermal control (heating during discharging). It was found that for 20 cycles without thermal control, the useful volumetric storage capacity decreased by 16%, and the amount of gas discharged by 10%. However, with the use of thermal management, efficiency losses have been reduced to 7% and 5%, respectively, due to more efficient ethane removal. However, thermal control showed no significant improvement in reducing the accumulation of hydrocarbons heavier than C_2_H_6_, which continued to displace methane and ethane from the system.

In this paper, we study the properties of active carbon synthesized from cheap peat raw materials, its properties for adsorption of methane and ethane, the two main components of natural gas, and their mixture adsorption by the methods of the ideal adsorbed solution theory (IAST) and molecular modeling. The cyclic mode of charging and discharging of a model scaled natural gas storage equipped with a synthesized adsorbent is also considered, and the effect of ethane accumulation and thermal control of the system on the higher heating value (HHV) of the discharged gas is shown.

## 2. Materials and Methods

### 2.1. Adsorbent and Adsorbed Gases

High purity methane (99.99%) and ethane (99.95%) produced by Linde Gas was used in adsorption data experiments. The properties of gases and their mixtures were determined using the CoolProp program [11] using NIST data.

High-moor peat of the wood group with a high degree of metamorphism was used as a raw material for the synthesis of the adsorbent.

The synthesis of nanoporous activated carbon ACPK from peat with a decomposition degree of more than 50% (H8 and higher on the Von Post scale) consisted of the stage of raw material preparation, carbonization without oxygen, thermochemical activation, washing, and drying. Thermochemical activation makes it possible to obtain a more uniform structure of narrow micropores.

The stage of preparation of raw materials included the processing and drying of peat, followed by grinding and sieving to separate the required fraction up to 2 mm in size. The carbonization stage was carried out in a muffle furnace without access to oxygen at a temperature of 800 °C with a heating rate of 10 °C/min. For activation, aqueous mixtures of carbonizate and KOH activator were prepared in ratios of 1:2, which were placed in a steel crucible and then in a furnace. Thermochemical activation was carried out at a temperature of 900 °C at a heating rate of 10 °C/min. After reaching the required temperature, the samples were kept for 1 h. After cooling the samples to ambient temperature, the samples were washed with distilled water to pH 8 and dried in an oven for 24 h at a temperature of 110 °C.

### 2.2. Characterization

The surface morphology and elemental composition of ACPK were examined by scanning electron microscopy (SEM) using a Quanta 650 FEG microscope (FEI Company, Hillsboro, OR, USA) equipped with an Oxford Energy Dispersive X-ray (EDX) detector operating at 15 kV accelerating voltage.

To study the phase composition of the studied adsorbent and the initial assessment of their physicochemical and adsorption properties, an X-ray powder diffraction study was carried out using an Empyrean Panalytical diffractometer in the range of scattering angles 2*θ* from 10 to 120°.

To evaluate the structural characteristics of the adsorbent, we used the small-angle X-ray scattering (SAXS) method on a SAXSess diffractometer (Anton Paar). Monochromatic radiation was obtained using a Cu-Kα filter (*λ* = 0.154 nm), and scattering was recorded on a two-dimensional Imaging Plate detector.

These methods provide a comprehensive understanding of the carbon framework and a primary understanding of the porous structure of the synthesized material ACPK. The detailed porous structure of ACPK was examined by N_2_ adsorption–desorption isotherms at 77 K, which were performed on a Quantachrome Autosorb iQ multifunctional surface area analyzer. The specific volume of micropores *W*_0_ (cm^3^·g^−1^), standard characteristic energy of adsorption *E*_0_ (kJ·mol^−1^), and effective half-width of micropores *x*_0_ (nm) were calculated by the Dubinin–Radushkevich (D-R) equation [12,13]. The Brunauer–Emmett–Teller (BET) method [14] using the criteria for microporous adsorbents [15] was also applied to evaluate the specific surface area, *S*_BET_ (cm^2^·g^−1^). The Kelvin equation [16] was used to calculate the specific surface area of mesopores, *S*_ME_ (cm^2^·g^−1^), respectively. The specific mesopore volume was calculated as *W_ME_* = *W*_S_ − *W*_0_, where *W*_S_ (cm^3^·g^−1^) is the total pore volume obtained from the nitrogen adsorption at the relative pressure *P*/*P*_s_ = 0.99. The pore size distribution function in ACPK was calculated using the Quenched Solid Density Functional Theory (QSDFT) developed for micro-mesoporous adsorbents [17].

### 2.3. Single-Component Adsorption

Single-component adsorption isotherms of C_2_H_6_ and CH_4_ were collected on purpose-designed adsorptions benches, the schemes of which are reported in the previous works [18,19,20]. Methane adsorption was studied at temperatures of 213, 243, 273, 293, 333, and 393 K at pressures up to 20 MPa. Ethane adsorption was studied at temperatures of 273, 293, 313, and 333 K at pressures up to 120 kPa. The selected measurement ranges cover the most common field of practical application of ANG systems. Before gas adsorption measurements, the degas process was carried out under vacuum at 250 °C for 6 h.

Adsorption at temperatures different from the experimental ones was determined by the property of the linearity of the isosteres in the ln *P*–1/*T* coordinates, which in turn were plotted from the experimental isotherms (at least three points). To expand the pressure range of the calculated isotherms, an additional extrapolation was carried out according to the equations that best describe the experimental adsorption isotherms: the Sips and the theory of volume filling of micropores (TVFM) equations [21]. The TVFM equation was used in the subcritical region, and the Sips equation in the supercritical. The study of the thermodynamic functions of the adsorption process was carried out using Bakaev’s approach [22,23,24], which is based on the method of changing variables to establish *a*, *P*, *T* (adsorption, pressure, and temperature, respectively) parameters of adsorption equilibrium in a wide range of pressures and temperatures, taking into account the nonideality of the gas phase in a thermodynamic system defined in accordance with the Guggenheim approach [25].

### 2.4. Mixture Adsorption Calculation

To determine the mixture adsorption of methane and ethane by the IAST method [26,27,28], the system of equations was solved:

(1) $$\frac{\pi }{RT}=\overset{P_{i}^{*}}{\underset{0}{\int }}\frac{a_{i}^{*}}{P_{i}}dP_{i}\;$$

(2) $$PY_{i}=P_{i}^{*}X_{i}\;$$

(3) $$\overset{N}{\underset{i=1}{\sum }}X_{i}=1\;$$

(4) $$\frac{1}{a_{\Sigma }}=\overset{N}{\underset{i=1}{\sum }}\frac{X_{i}}{a_{i}^{*}}\;\;,$$

where π is the spreading pressure, (Pa); *T* is the temperature, (K); *R* is the universal gas constant, (J/(mol K)); $a_{i}^{*}$ is the adsorption of the pure component *i* of the mixture, (mmol/g), at *T* and the equilibrium pressure of the gas phase $P_{i}^{*}$ (Pa), which corresponds to π; $X_{i}$ is the mole fraction of component *i* in the adsorbed phase; $Y_{i}$ is the mole fraction of component *i* in the gas phase; $a_{\Sigma }$ is the adsorption of the mixture in mmol/g at its equilibrium pressure *P*, (Pa).

The integral reduced (to the mass of the adsorbent) enthalpy of the mixed adsorbate was calculated as the sum of the integral reduced enthalpies of the adsorbate components at spreading pressure π and, accordingly, $a_{i}^{*}$, corrected for the actual filling of the pore with this component $a_{i}$:

(5) $$h_{a}=\overset{N}{\underset{i=1}{\sum }}\frac{h_{i}\left(a_{i}^{*},T\right)}{a_{i}^{*}}a_{i}=\overset{N}{\underset{i=1}{\sum }}h_{i}\left(a_{i}^{*},T\right)\frac{X_{i}\cdot a_{\Sigma }}{a_{i}^{*}}\;\;,$$

where $h_{i}$ is the integral reduced (to the mass of the adsorbent) enthalpy of the pure component *i* of the adsorbate at adsorption $a_{i}^{*}$ and temperature *T*, (J/kg).

### 2.5. Molecular Modeling

The adsorption of a methane–ethane mixture was modeled by molecular dynamics using the Dynamic software module from the Tinker molecular modeling software package [29]. To reduce the cost of computer time, the universal atom–atom force field OPLSAA was used [30], which simulates the total interaction potential. It was shown in [31] that among the existing universal potentials, this force field is best suited for describing model adsorption systems consisting of carbon structures and hydrocarbon molecules.

The simulation was carried out in the canonical (*N*, *V*, *T* are the number of molecules, volume, and temperature, respectively) ensemble. The simulation cell was a parallelepiped. Its height corresponded to the width of the pore plus 2 radii of carbon atoms. Micropores with a width in the range from 0.6 to 1.8 nm with a step of 0.2 nm were studied. The selected range of micropore widths corresponds to the most common widths for industrial carbon adsorbents. The walls of micropore are formed by single-layer graphene. The side faces of the simulation cell were 10 nm. The edges of the simulation cell are limited by periodic boundary conditions. The temperature of the numerical experiment was 293 K. Andersen’s thermostat was used to thermostat the simulation system [32]. Studies of the molecular dynamics trajectory were carried out in the time interval 2 × 10^−9^ s. The elementary step of integrating the equation of motion was 10^−15^ s. Averaging of the system parameters for processing the results of a numerical experiment was carried out every 10^−12^ s. The time to reach the equilibrium states of the studied systems was estimated from the change in the total energy with time; it was no more than 10^−9^ s.

### 2.6. Adsorption Storage

The paper considers a model of an adsorption storage with a volume of 1 m^3^, limited by an aluminum shell. The calculation uses 5005-H18 alloy, which has average characteristics for aluminum alloys and is comparable in strength to popular stainless steels. With a shell mass of 250 kg or 500 kg, the maximum safe pressure inside is 5.0 and 10.0 MPa, respectively, however, taking into account possible temperature changes during gas storage, charging should be limited to pressures of 3.5 and 7.0 MPa, respectively. The storage is completely filled with a monolith adsorbent formed from ACPK active carbon and a polymeric binder with mass fractions of 95% and 5%, respectively. The density of the monolith adsorbent in the dried state is 720 kg/m^3^ (corresponds to the indicators achieved in practice). The storage model is lumped, zero-dimensional, with a uniform distribution of temperatures, pressure, and adsorption of each component of the mixture.

**Figure 1:**
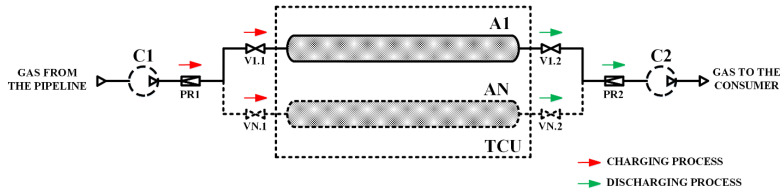
Scheme of the adsorption storage. C1, C2–natural gas compressors; A1…AN–ANG storage systems (adsorption tanks); TCU–thermal control unit; V1.1…VN.1 and V1.2…VN.2–stop valves; PR1-2–pressure regulators.

A simplified scheme of the adsorption storage is shown in Figure 1. The storage can consist of several tanks A1…AN, charging up to a pressure of 3.5 or 7.0 MPa (before the pressure regulator PR1) using a compressor C1 or directly from the gas pipeline, while the temperature of the supplied gas is constant (in all calculations 293 K). Gas is supplied to the consumer at a pressure of 0.1 MPa (after the PR2 regulator); if necessary, the pressure of the gas supplied to the consumer is increased using the C2 compressor. Constant pressure at the inlet (before PR1) and outlet (after PR2) allows us to assume that no technical work is done on the system, and the change in the enthalpy of the adsorption storage occurs due to the enthalpy of the inlet or outlet gas and heat supply or removal. The model is based on the assumption that there is no natural heat exchange with the environment: heat flow only occurs forcibly and is controlled using the TCU–thermal control unit. In practice, thermal control is provided using built-in heat exchangers [22,33,34] or gas circulation through the adsorbent bed with cooling/heating in an external heat exchanger [35,36,37].

The paper analyzes the cyclic charging and discharging processes in the adsorption storage in three different modes:

(a) in “isothermal” mode, which can be oriented as a base case;
(b) in a mode without heat exchange with the environment, which can be considered conditionally “adiabatic” (if we do not take into account the mass transfer due to the charging and discharging of gas);
(c) in “thermo-controlled” mode with cooling at gas charging and heating at gas discharging. In terms of temperature change, this mode is opposite to the “adiabatic” mode.

Cycling calculations are based on the heat balance of the system and the mass balance for each component.

The mass of each component of the mixture in the adsorption storage is determined by the expression, (kg):

(6) $$M_{i}=\left(\rho_{p}\left(1-x_{b}\right)a_{i}\mu_{i}+Y_{mi}\varepsilon \rho_{g\Sigma }\right)V_{s}\;,$$

where $\rho_{p}$ is the packing density of the adsorbent, (kg/m^3^); $x_{b}=0.05$ is the mass fraction of the polymer binder; $\mu_{i}$ is the molar mass of component *i*, (kg/mol); $\rho_{g\Sigma }$ is the density of the gas phase as a mixture, (kg/m^3^) at pressure *P* and temperature *T* in storage; ε is the porosity, the fraction of space free for the gas phase; $Y_{mi}$ is the mass fraction of component *i* in the gas phase; $V_{s}$ is the volume of adsorption storage, (m^3^).

The enthalpy of the entire storage is the sum of the enthalpies of its individual components, (J):

(7) $$H_{\Sigma }=\left(h_{a}\rho_{p}\left(1-x_{b}\right)+h_{g\Sigma }\varepsilon \rho_{g\Sigma }\right)V_{s}+H_{b}+H_{s}\;,$$

where $h_{g\Sigma }$ is the specific enthalpy of the gas phase as a mixture, (J/kg); $H_{b}$ is the enthalpy of the binder, (J); $H_{s}$—is the enthalpy of the storage metal shell, (J).

## 3. Results and Discussion

### 3.1. Characterization

**Figure 2:**
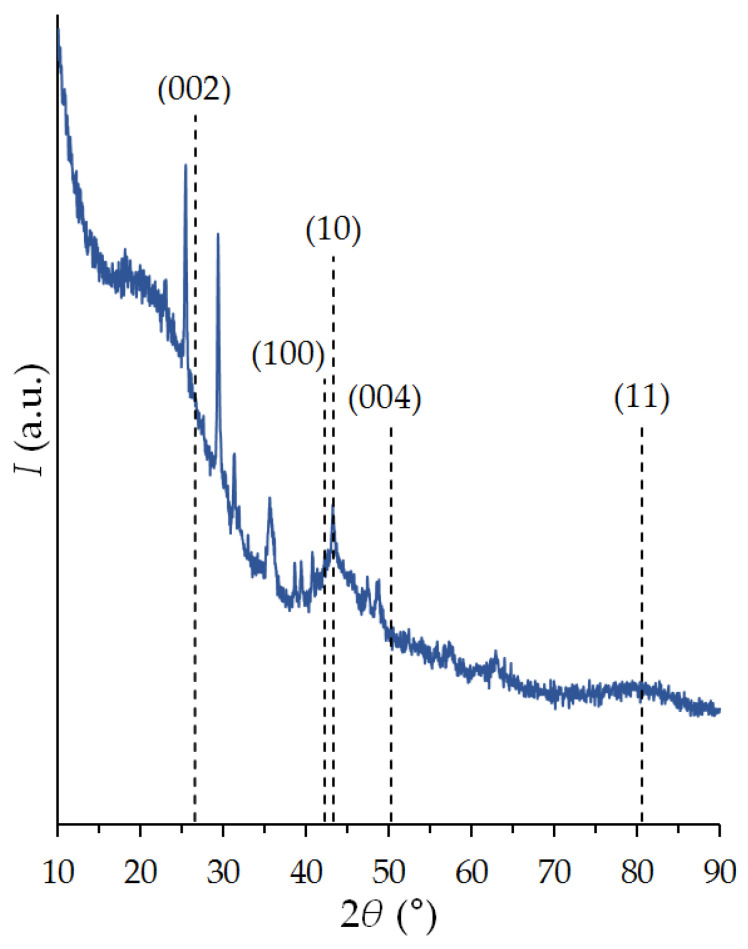
X-ray diffraction pattern of the carbon adsorbent ACPK. Graphite reflections are (002), (10), (100), (004), and (11).

ACPK carbon adsorbent is made from highly decomposed peat, the content of carbon and mineral impurities in which can vary over a wide range. As a result, the adsorbent is heterogeneous in content. Part of the carbon remained in its original form of large nanocrystals of ordered graphite, for which narrow reflections (002), (10), (100), (004), and (11) are characteristic, Figure 2. An increase in the background to small angles indicates the content of high-carbon radicals of the amorphous phase in the structure of the adsorbent. In addition to reflexes related to graphite deposits, a number of extraneous peaks are also observed, indicating the presence of mineral inclusions.

**Figure 3:**
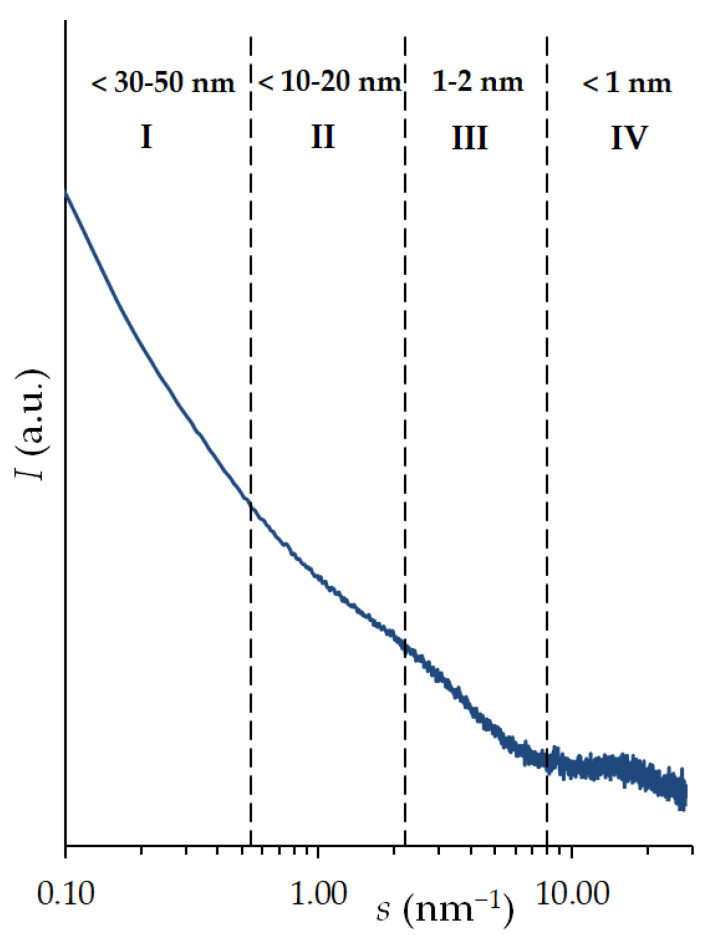
Dependence of the small-angle X-ray scattering intensity on the carbon adsorbent ACPK on the scattering vector at small and medium angles. I–IV are the characteristic X-ray scattering data areas used to obtain the structural parameters of the adsorbents.

Based on the experimental data of SAXS (Figure 3), for the region of inhomogeneities III (1–2 nm), which correspond to scattering in micropores, the radius of gyration (inertia) *R*_G_ was calculated using the method in [38]. This integral parameter conditionally characterizes the average sizes of micropores, and thus is most indicative in the analysis of the porous structure of carbon adsorbents [39]. Based on the obtained values of *R*_G_, the sizes of model micropores of different shaped adsorbents were determined: spherical *R*_S_ and cylindrical *R_T_*. The results of determining the parameters of the model adsorbent pores and gyration radius are shown in Table 1.

**Figure 4:**
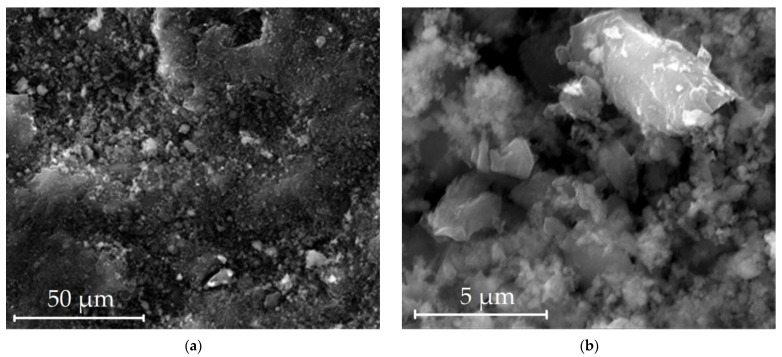
Electron microscopy images of the surface of the adsorbent from peat raw material ACPK: 50 µm (**a**); 5 µm (**b**).

Pictures of the surface by scanning electron microscopy (SEM) are shown in Figure 4. The structure of the adsorbent has a granular type, and an inhomogeneous appearance in shape and size. In places, grains of a layered type are observed, similar to carbon graphite.

The presence of a large number of mineral impurities on the surface of the adsorbent is probably due to the fact that the raw in the form of fossil peat and coal dust contains a significant proportion of various elements. High content of impurities, up to 13.3% wt. (Table 2), may qualitatively indicate that the contribution to the energetics of adsorption of peat coals is made by surface chemistry, and not only by the width of micropores and their size distribution.

**Figure 5:**
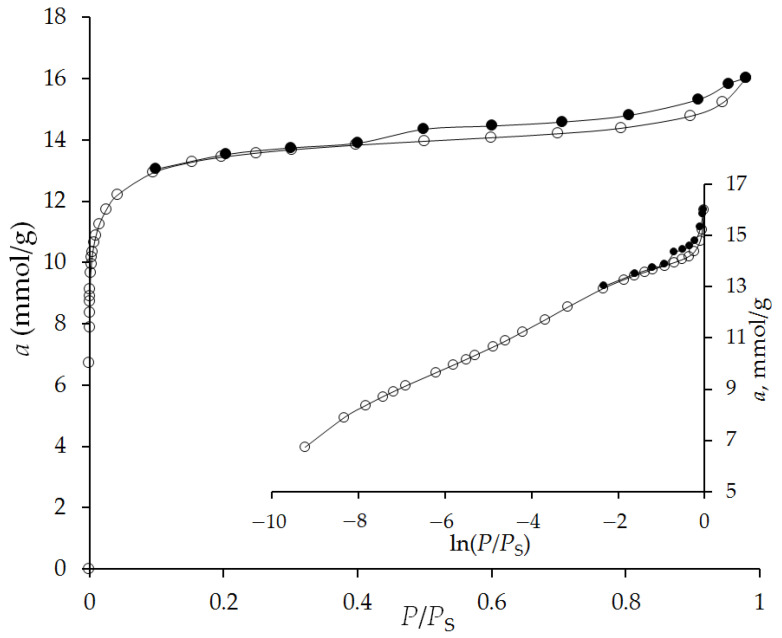
Adsorption (light symbols) and desorption (solid symbols) isotherms of nitrogen on the microporous carbon adsorbent ACPK at 77 K in linear and semi-logarithmic coordinates. Lines–spline approximation.

Adsorption–desorption isotherms of standard nitrogen vapor at 77 K of the studied adsorbent are shown in Figure 5. As follows from Figure 5, the isotherm in the coordinates *a = ƒ(P/P*_S_*)* has an L-shaped form of type I [40] in the initial region of the isotherm up to 0.3 *P/P*_S_, which indicates the presence of a developed volume of micropores in the porous structure of the adsorbent. At pressures close to the saturated vapor pressure, the adsorption–desorption isotherms show a capillary-condensation hysteresis loop of the H4 type [40], which is characteristic of a mesoporous structure.

Table 3 presents the parameters of the porous structure of the ACPK adsorbent. As follows from Table 3, the mesopore volume of the adsorbent is about 25% of the total pore volume, and the mesopore surface is 40 m^2^/g. Thus, the adsorbent contains a significant proportion of mesopores, the adsorption of gases in which must be taken into account.

**Figure 6:**
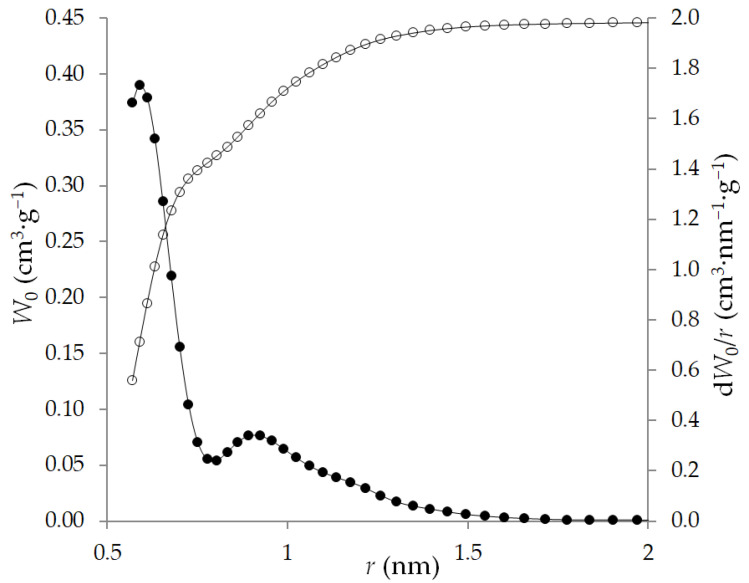
The QSDFT cumulative *W*_0_ (light symbols) and differential d*W*_0_/*r* (dark symbols) pore volume distributions calculated for ACPK from the nitrogen adsorption at 77 K for a sphere pore model.

To estimate the size distribution of micropores, we used the approach of the density functional theory QSDFT for a spherical pore model as the most probable pore model for a given adsorbent according to X-ray diffraction data. The distribution curves of micropores by size d*W*_0_/d*d* = *f*(*d*) are shown in Figure 6. The maximum on the micropore size distribution curve corresponds to *r_max_* = 0.56 nm.

The pore parameters determined from the results of XRD (Table 1) coincide with the determination by the TVFM [41] (Figure 5, Table 3) and are close to the pore size distribution determined by the QSDFT method (Figure 6, Table 3). In this case, the adsorbent has a second, less pronounced maximum on the distribution curve, with a radius corresponding to the distribution maximum of 0.92 nm. The ratio of pore volumes for the two distribution maxima is about 75/25% for narrow and wide pores, respectively.

### 3.2. Equilibrium Adsorption Tests

**Figure 7:**
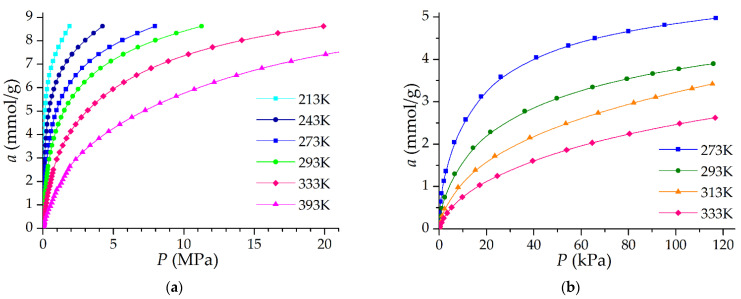
Adsorption isotherms of methane (**a**) and ethane (**b**) on the ACPK adsorbent. Symbols are an experiment. The lines are a spline approximation.

Methane adsorption on ACPK was measured in the temperature range from 213 to 393 K, and ethane in the temperature range from 273 to 333 K. The results are shown in Figure 7. The isotherms of both gases are type I isotherms [40]. At a pressure of 3.5 MPa and 293 K, the adsorption of methane on the synthesized adsorbent ACPK is 6.55 mmol/g or 10.5% wt., and at a pressure of 7.0 MPa and 293 K, respectively, 7.80 mmol/g and 12.5% wt. These adsorption properties make it possible to use this adsorbent for the purpose of methane (natural gas) storage, but under the condition of a high packing density of the adsorbent, for example in the form of monoliths [34,42,43,44,45].

**Figure 8:**
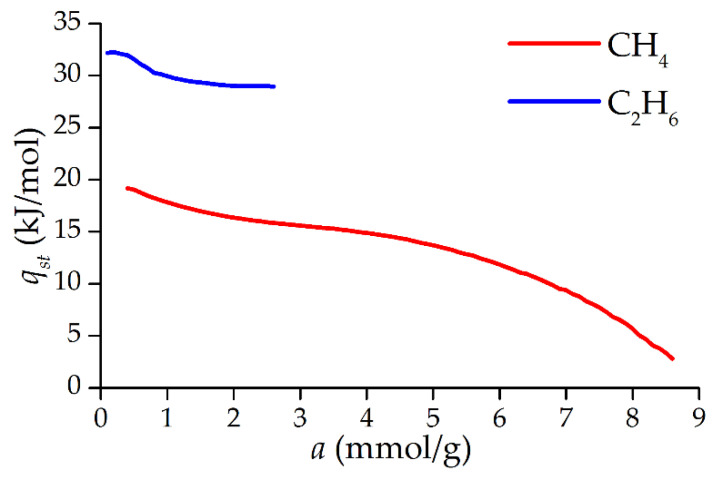
Differential molar isosteric heat of adsorption of methane and ethane on the microporous carbon adsorbent ACPK at a temperature of 293 K, determined from experimental data.

Figure 8 shows the dependence of the differential molar isosteric heat of adsorption of methane and ethane on the nanoporous carbon adsorbent ACPK at a temperature of 293 K. The plotting area for C_2_H_6_ is limited by the area of reliable experimental measurements of adsorption with the possibility of isostere linearization by at least three points. Heat is determined without taking into account adsorption-stimulated and thermal deformation. The value of carbon adsorbents deformation under the described conditions does not exceed 0.3–0.4%, while the contribution of deformation to the heat of adsorption at a temperature of 293 K does not exceed 2–5% [23,46,47]. The absence of an abrupt jump in the heats of adsorption in the initial region of filling indicates a weak inhomogeneity of the adsorbent sorption surface. A barely noticeable extremum at 0.2 mmol/g is observed on ethane, but this is probably due to experimental errors due to the proximity to the boundary of the measurement region. Otherwise, the dependences of the heats of adsorption of methane and ethane in the studied area repeat the shape of each other.

### 3.3. Mixture Adsorption

**Figure 9:**
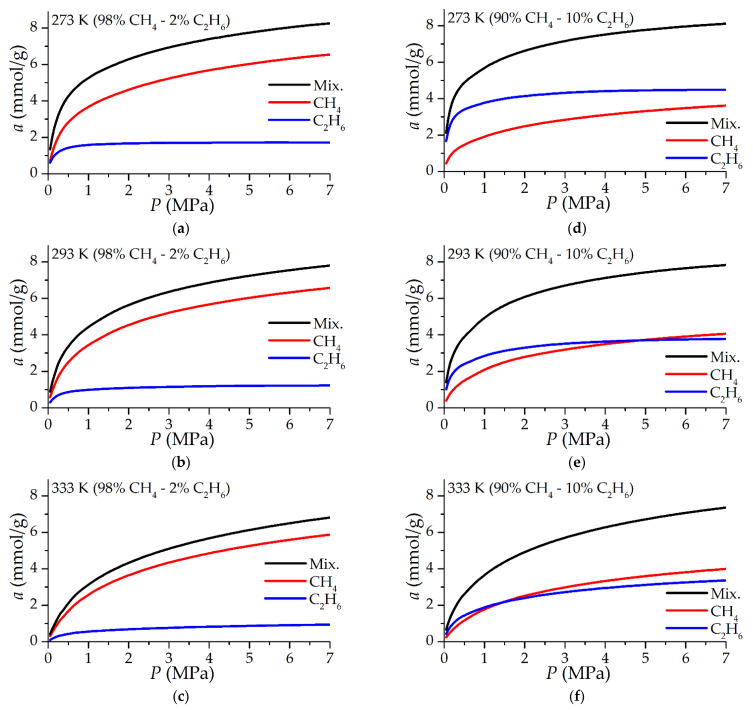
Equilibrium adsorption of a binary mixture of methane and ethane depending on the pressure of a gas mixture with a molar content of 2% (**a**–**c**) and 10% (**d**–**f**) ethane at temperatures of 273 K (**a**,**d**), 293 K (**b**,**e**), 333 K (**c**,**f**). The calculation method is IAST using experimental isotherms of pure components.

The IAST results of determining the adsorption of a mixture and individual components in a mixture of CH_4_ and C_2_H_6_ are shown in Figure 9. With a content of 2% mol. ethane in the gas phase, its contribution to the total adsorption is much more significant: from 14.7% mol. at 3.5 MPa and 333 K up to 23.8% mol. when the temperature decreases to 273 K. At the same time, methane remains the main adsorbed gas in the entire considered pressure range from 0.05 to 7 MPa. At a content of 10% mol. ethane in the gas phase, the situation is reversed: ethane is adsorbed in even larger quantities than methane at a temperature of 273 K (59.5% mol. of ethane in the total adsorption at 3.5 MPa) to approximately equal adsorption with methane at a temperature of 333 K.

It is obvious that such active absorption of ethane can adversely affect the active capacity of the adsorption storage due to the fact that ethane is less efficiently desorbed upon depressurization compared to methane. On the other hand, as can be seen from Figure 9, the total molar adsorption of the mixture varies slightly with a change in the ethane content in the gas phase, however, the higher heating value of 1 mol of ethane is 75% higher than that of methane. Thus, the energy capacity of the adsorption storage increases with an increase in the ethane concentration: at a pressure of 3.5 MPa and 293 K, the higher heating value of the adsorbate at an ethane concentration of 10% mol. in the gas phase is 8.57 MJ per 1 kg of adsorbent, while in the case of pure methane this heat is 5.84 MJ/kg–1.47 times less–while the higher heating value of the gas phase differs by only 7.5%. In some cases, this effect can be useful; for example, by integrating the storage into a flowing gas pipeline network: the storage capacity will increase significantly due to the capture of ethane and heavy hydrocarbons from the flow. However, the extraction of this excess energy is complex due to the difficulty of extracting ethane (and other heavy hydrocarbons, if present in natural gas). The advantages and disadvantages of the presence of impurity hydrocarbons in natural gas depend on the form of the gas charging and discharging processes organization.

### 3.4. Molecular Modeling

For a more detailed understanding of the mechanism of mixture adsorption of methane and ethane, a numerical simulation in model pores of a carbon adsorbent of various widths *H_S_* from 0.6 to 1.8 nm with a step of 0.2 nm was performed using the molecular dynamics method. For the study, a slit-like micropore model was used as it is one of the most common models for the formation of pores in carbon adsorbents. [48,49]. Carbon adsorbents, in general, consist of an ordered part of carbon crystallites and an amorphous part of high-carbon radicals [50]. The ratio of the amorphous and crystalline parts of carbon is determined by the raw and carbonization conditions. When using organic carbon-containing raw materials, the structure of crystallites resembles graphite, but they themselves are packed less regularly in the volume of carbon. At the same time, an increase in activation leads to a decrease in the fraction of crystallites and an increase in the amorphous fraction. The amorphous part may contain micropores of various shapes, including those close to spherical. According to the results of the adsorbent structure study (Table 1 and Table 3 and Figure 6), the ACPK adsorbent contains a significant part of amorphous carbon with a pore shape close to spherical. On the other hand, X-ray diffraction analysis (Figure 2) revealed the presence of peak characteristics of graphite-like microporous structures. Thus, the use of the slit-like micropores of graphite-like crystallites model of a carbon adsorbent in a numerical simulation is legitimate. A numerical experiment was carried out for a mixture 95% CH_4_ and 5% C_2_H_6_. The temperature of the experiments was 293 K. The concentration of the mixture for the study was selected based on the data on the average concentration of C_2+_ hydrocarbons.

**Figure 10:**
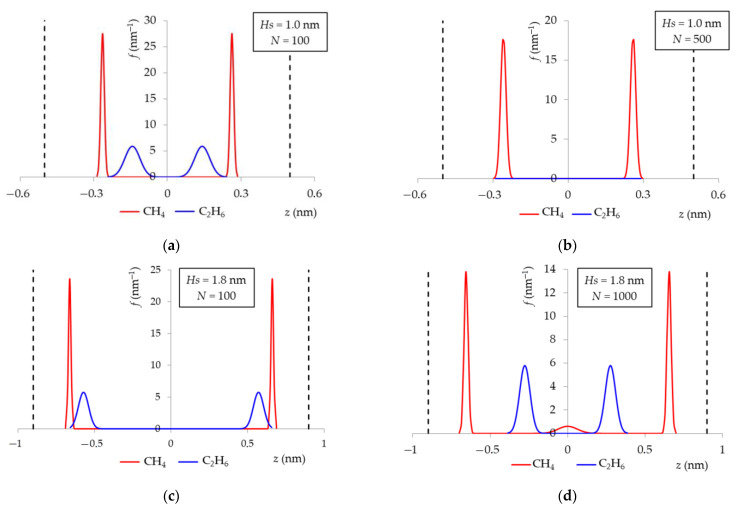
Probability density of the mass center location of a methane and ethane molecule in a model pore of a carbon adsorbent with a width of *H_S_* = 1.0 nm (**a**,**b**) and 1.8 nm (**c**,**d**) for different numbers of molecules *N* in the simulation cell, corresponding to the region of averages (**a**,**c**) and maximum (**b**,**d**) fillings of micropores. The number of molecules of the mixture *N* (the ratio of the molecules number of methane and ethane is 95:5) in the modeling system, pcs: 100 (**a**); 500 (**b**); 100 (**c**); 1000 (**d**). The temperature is 293 K. The ordinate axis intersects the abscissa axis at point *z* = 0, corresponding to the symmetry plane of the pore. The dotted line is the boundary of the model micropore.

Figure 10 shows the dependencies of the probability of the mass center location of a methane or ethane molecule in a pore for various pore widths and their filling. In the process of adsorption, for narrow pores 0.6, 0.8, and 1 nm wide, the concentration displacement of ethane is shown. For pores with a width of 1 nm, close to the size of ACPK micropores according to X-ray diffraction analysis (Table 1) and the first maximum on the QSDFT pore size distribution curve (Figure 6), the mixture is almost completely sorbed in the pore at low fillings (up to 100 molecules in the modeling system), forming molecular complexes near the graphene walls, Figure 10a. Figure 10a shows that the centers of mass of ethane molecules are shifted relative to the centers of mass of methane molecules, which can be explained by the fact that linear ethane molecules tend to occupy a perpendicular position to the micropore walls. With an increase in the number of molecules in the simulation system, methane molecules completely fill the molecular complexes located in the potential minima of the model micropore near the graphene surface, displacing ethane molecules outside the pore, Figure 10b.

For micropores with a width of more than 1.2 nm, the effect of concentration displacement is not observed. Figure 10c,d show the simulation results for a wide model micropore with a width of 1.8 nm, which is close in value to the wide ACPK pores corresponding to the second distribution maximum in Figure 6. At low fillings, the behavior of adsorbate molecules is similar to that observed in narrow pores, but at high fillings, ethane molecules are present in the micropore, forming a “layered” structure with methane molecules, while ethane molecules are displaced from molecular complexes closer to the center of the micropore.

**Figure 11:**
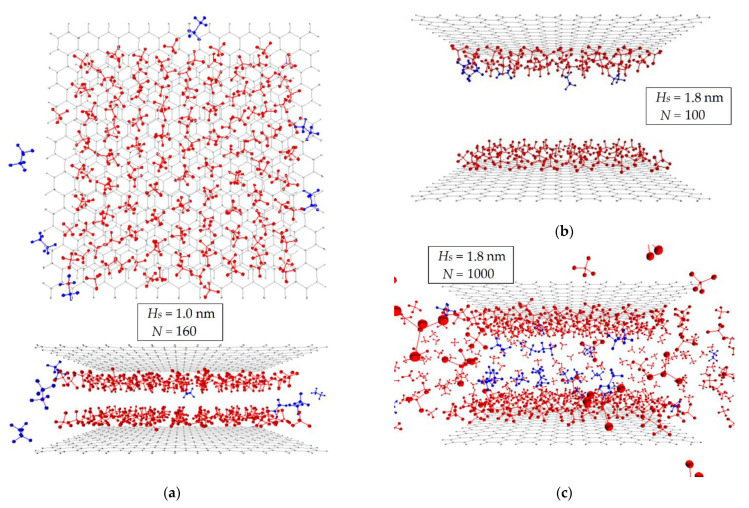
Instantaneous snapshot of the molecular dynamics trajectory of a simulation system consisting of two graphenes at a distance of *H_S_* = 1.0 nm (**a**) and 1.8 nm (**b**,**c**) at different numbers of *N* molecules (the ratio of the number of molecules of methane and ethane is 95:5) in simulation cell: 160 (**a**); 100 (**b**); 1000 (**c**). The bonds of atoms forming graphene are indicated by solid lines. Atoms are shown as unscalable red (methane) and blue (ethane) spheres.

Figure 11 shows instantaneous snapshots of the molecular dynamics trajectory of the simulation system for micropores of different widths. These images clearly demonstrate the dependences shown in Figure 10, while expanding our understanding of the behavior of methane and ethane molecules in a micropore. Thus, in a narrow micropore 1.0 nm wide, sorbed ethane molecules are located closer to the edges of graphenes, Figure 11a, probably due to the predominant interaction of methane molecules with each other in the field of dispersion forces of the adsorbent than the interactions of methane–ethane. As a result, ethane molecules are forced out of the narrow micropore.

In the case of a wide pore 1.8 nm wide, the situation is different: Figure 11b shows that methane and ethane molecules together form molecular complexes in accordance with Figure 10c. With further filling of a wide micropore, ethane is not completely displaced from the pore, Figure 11c: in the center of the micropore, the density of molecules is low, which allows two more layers of ethane molecules and even a third layer of methane between them to be accommodated, Figure 10d.

**Figure 12:**
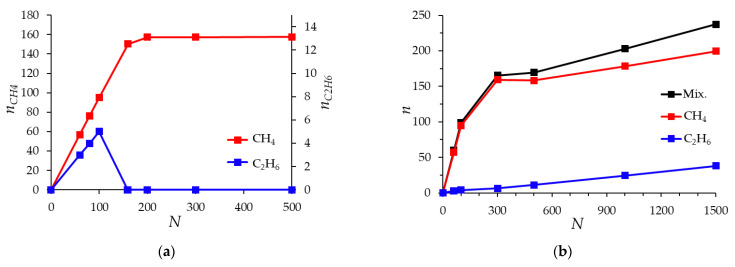
Dependence of the number of adsorbed molecules *n* of methane CH_4_ and ethane C_2_H_6_ and their total number (Mix.) on the number of molecules in the simulation cell *N* (ratio of the number of molecules of methane and ethane 95:5) for a pore with a width of 1.0 nm (**a**) and 1.8 nm (**b**). The temperature is 293 K.

Figure 12 shows the dependence of the number of adsorbed methane and ethane molecules on the number of molecules in the ratio of 95% methane and 5% ethane in simulation cells with a width of 1.0 nm and 1.8 nm at a constant temperature of 293 K, the molecular adsorption isotherm of the mixture.

In a narrow micropore 1.0 nm wide, Figure 12a, in the region of up to 100 molecules (95 methane molecules and 5 ethane molecules), a classical increase in the amount of adsorbed methane and ethane is observed in the system. Moreover, almost all ethane molecules and most of the methane molecules in this region from the free phase enter the micropore. With an increase in the number of molecules in the simulation system, the number of ethane molecules in the pore gradually decreases due to its displacement by methane. At 160 molecules of the mixture (an instantaneous snapshot was presented in Figure 11a) in the simulation system, ethane is completely displaced by methane from micropores: an interesting behavior is observed that if there are ethane molecules in the free phase (analogue of the gas phase), they are not in the adsorption pore. This behavior is probably explained by the difference in size and shape of methane and ethane molecules. Small and spherically symmetric methane molecules fill the micropore into two adsorption layers, forming a more energetically favorable state of the adsorbate than ethane molecules, which, due to their linear shape, tend to occupy a perpendicular position in the narrow micropore and thereby prevent the formation of the second adsorption layer. Figure 12b shows the results of modeling the mixture adsorption in wider pores, using the example of a micropore 1.8 nm wide. In this case, ethane adsorption continuously increased as the number of molecules in the system increased. From the previously considered Figure 10 and Figure 11, it is already known that ethane is not completely replaced by methane in a wide pore.

**Figure 13:**
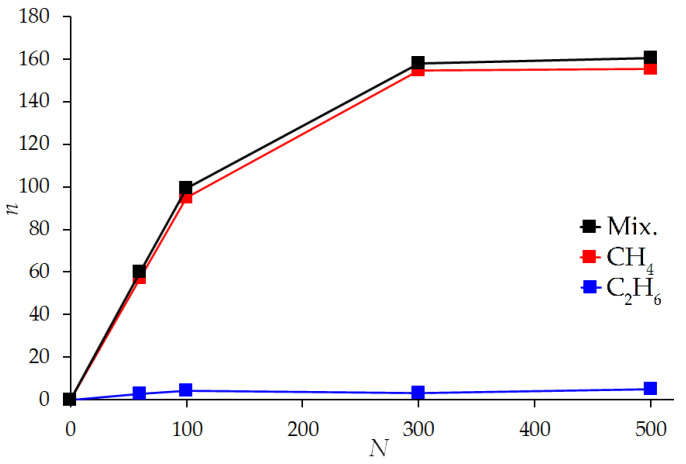
Integral dependence of the number of adsorbed molecules *n* of methane CH_4_ and ethane C_2_H_6_ and their total number Mix. on the number of molecules *N* (ratio of the number of molecules of methane and ethane 95:5) in an array of simulation cells, quantitatively corresponding to the pore size distribution of the ACPK adsorbent. The temperature is 293 K.

The obvious differences between the molecular isotherms shown in Figure 12 and the adsorption isotherms of the mixture calculated using the IAST model (Figure 9) are explained by the fact that the IAST method is based on the isotherms of the pure components, which are in fact integral isotherms over all pores of a real adsorbent with a wide pore size distribution. Figure 13 shows an attempt by the authors to evaluate the integral adsorption isotherm of a mixture obtained by summarizing molecular isotherms for pores of various widths, the weight contribution of which is determined by the share of pores of the corresponding width in the pore size distribution defined by the QSDFT method (Figure 6) for the ACPK adsorbent. As can be seen from Figure 13, the integral molecular adsorption isotherms of the mixture and their components behave similarly to the isotherms calculated using the IAST model: thus, the adsorption of ethane in a wide range of fillings (from 100 to 500 molecules) changes relatively weakly, similar to the saturation of ethane in Figure 9. This is due to the simultaneous decrease and increase in the amount of sorbed ethane in pores of various sizes. A direct comparison of the integral molecular isotherm and IAST isotherms is difficult due to the correct consideration of the free (gas) phase, as well as differences in the pore structure of the real and model adsorbent, due to the presence of a significant fraction of the amorphous carbon phase in the structure of the real adsorbent.

Despite the fact that the IAST model and the molecular model can produce results similar in form, it is obvious that some molecular features of the filling of micropores of different sizes with certain substances cannot be taken into account in principle by the IAST model, even when applying this model to a specific model micropore. For example, the case of a slit-like micropore 1.0 nm wide: the adsorption of pure methane and ethane in it is not equal to zero, but the mixture adsorption, according to the results of molecular modeling, can lead to the complete displacement of one of the components, which is not observed in the IAST model.

### 3.5. Cycles in Adsorption Natural Gas Storage

To assess the influence of thermal effects and the presence of impurities (ethane in a binary mixture with methane), modeling of cyclic processes of gas charging and discharging was performed using the IAST method. Idle time and gas storing processes have been eliminated, charging starts immediately after discharging, and vice versa. In the initial state, the adsorption storage is filled with pure methane at a pressure of 0.1 MPa and a temperature of 293 K. The storage is charged up to 3.5 or 7.0 MPa. The supplied gas has a pressure of 3.5 or 7.0 MPa, respectively, and a temperature of 293 K. Gas leaves the storage under variable conditions corresponding to the conditions in the storage. Since in the considered zero-dimensional model (with lumped parameters) all processes are in equilibrium and there is no natural heat exchange with the environment, the time factor does not play a significant role. The time scale is replaced by the gas exchange scale: the total mass of gas entering and exiting the storage. If we assign certain flow rates for gas charging and discharging, then such a gas exchange scale is definitely translated into a time scale, but this does not play a role for the analysis of cycles.

**Figure 14:**
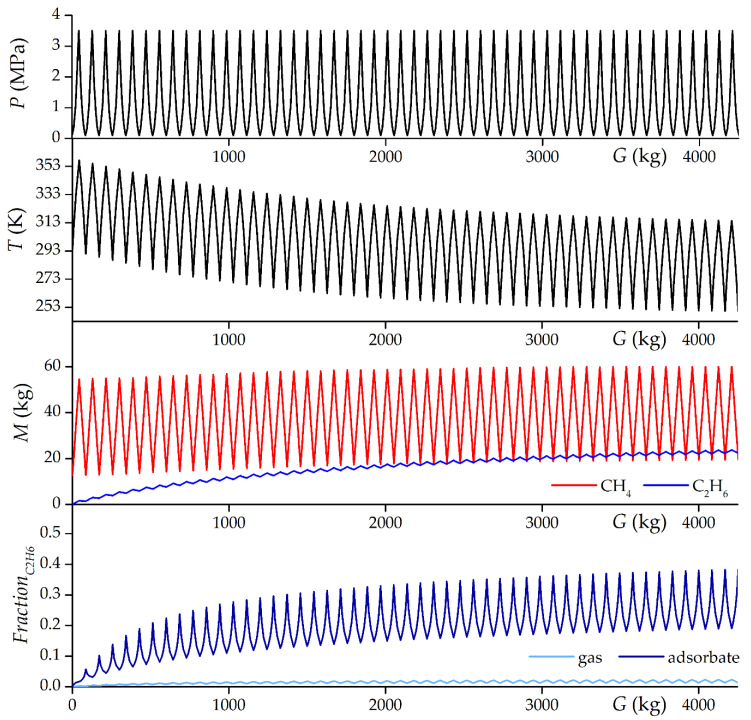
Dependencies of pressure, temperature, and mass of methane and ethane in the adsorption storage with a volume of 1 m^3^ based on ACPK adsorbent and the mole fraction of ethane in the gas and adsorbed phases in the “adiabatic” cyclic charging/discharging process and supplied gas with 98% mol. methane and 2% mol. ethane using the IAST simulation results from the cumulative gas exchange since the beginning of the simulation. The first 50 cycles are shown.

Figure 14 shows the results of modeling the first 50 cycles of “adiabatic” (without heat exchange with the environment and thermal control) charging up to 3.5 MPa with gas containing 98% mol. methane and 2% mol. ethane and gas discharging up to 0.1 MPa from storage. The gradual cooling of the storage under “adiabatic” conditions is explained by mass exchange with the external environment: the incoming gas at a temperature of 293 K is colder than the outgoing gas in the first cycles, which ultimately leads to the stabilization of the storage temperature below the initially set 293 K. Despite the very low content of ethane in the inlet gas, it accumulates in the system until the outlet gas has, on average, the same concentration of ethane as the inlet gas. Therefore, in the first approximation, the effect of ethane can be estimated from the equilibrium adsorption isotherms of the mixture shown in Figure 9. However, it should be taken into account that the molar fraction of ethane changes during the process. The share of ethane in the adsorbate after 50 cycles reaches 41.5% mol. at the end of discharging (at the minimum pressure in the system) and about 21.5% mol. at the end of charging (at maximum pressure in the system). At the end of 50 cycles shown in Figure 14, the periodic mode is not yet steady. The periodic mode can be considered established approximately around 110 cycles of adiabatic charging and discharging, when the difference in the amount of charged and discharged ethane differs by less than 1%, and this error becomes comparable with the mathematical errors of the model itself.

To assess the effect of impurities in fuel storage systems, it should be taken into account that both considered gases are combustible and targeted. Therefore, the influence of impurities should be assessed from the position of loss in the energy capacity of the storage. The same approach makes it possible to take into account the thermal effects of adsorption and desorption, which are expressed in a lower active storage capacity. The usual approach to assessing the active capacity of an adsorption storage in terms of volume (m^3^ of gas per m^3^ of volume) is incorrect, since 1 m^3^ of ethane under normal conditions (101,325 Pa and 293.15 K) has the higher heating value by 76% more than 1 m^3^ of methane. The assessment by weight can be considered justified: the difference in the combustion energy of 1 kg of methane and ethane is only 7%, which with ethane content up to 10% mol. leads to a mass combustion energy range of 54.9–55.5 MJ/kg.

**Figure 15:**
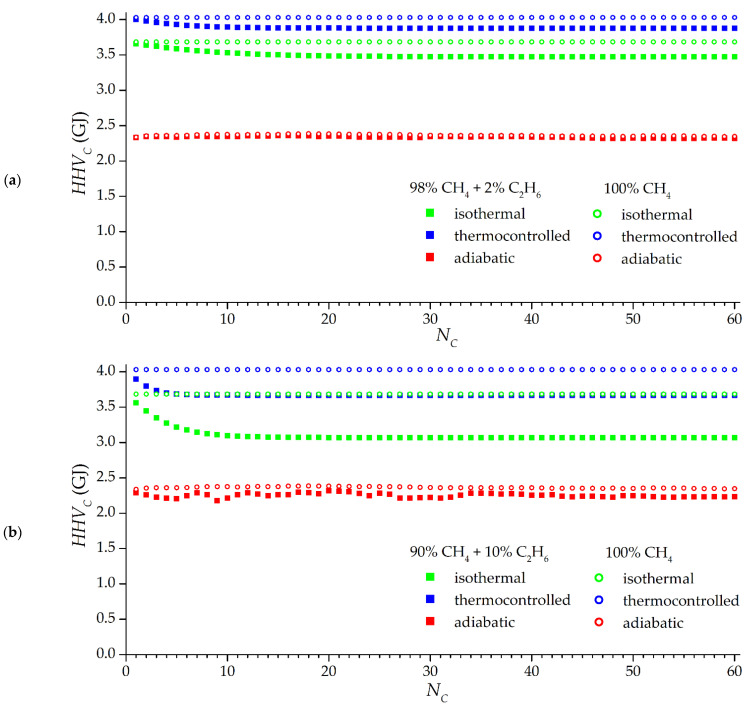
Dependence of the higher heating value of the mixed gas leaving for one cycle from a storage with a volume of 1 m^3^ based on the ACPK adsorbent, on the cycle number. Filled dots correspond to mixtures: 98% mol. methane and 2% mol. ethane (**a**); 90% mol. methane and 10% mol. ethane (**b**). Empty points correspond to pure methane.

Figure 15 shows the dependencies of the higher heating value of the discharging gas by cycles for various types of charging up to a pressure of 3.5 MPa and discharging up to 0.1 MPa: “adiabatic”, “isothermal”, which is not ideal, but convenient as a reference point, and “thermocontrolled”, the opposite of adiabatic, i.e., with cooling when charging up to 293 K and heating when discharging gas up to 333 K. The adiabatic mode significantly reduces the active energy capacity of the storage: by 27–33% compared to the isothermal process with the same composition of the supplied gas; this is the influence of the thermal effects of adsorption and desorption. Surprisingly, in the adiabatic mode, the accumulation of ethane has practically no effect on the active energy capacity of the storage (compared to operation on pure methane): losses in methane are compensated over time by a large amount of ethane released. The “harmful” accumulation of ethane is more noticeable in isothermal and thermocontrolled modes: over time, the active energy capacity of the storage decreases to a certain steady-state value. In isothermal mode 2% mol. the concentration of ethane in the gas supplied leads to a loss of efficiency of 5.7%, and at a concentration of 10% mol. losses already 16.6%. The accumulation of ethane has a greater effect on the isothermal mode.

The advantages of the thermocontrolled mode in the energy storage capacity compared to the isothermal mode depend on the composition of the supplied gas: with a large number of cycles, the advantage is 9.4% on pure methane (due to a higher temperature at the end of the discharging process), 11.7% at 2% mol. fraction of ethane in the gas supplied to the system, and 19.5% at 10% mol. Fraction of ethane, i.e., the temperature-controlled mode allows for more efficient removal of ethane and contributes to a reduction in its accumulation. It can also be seen from Figure 15 that thermocontrolling reduces the duration of reaching a stable periodic mode compared to the isothermal mode, while the opposite adiabatic mode can be unstable for quite a long time. The greater the proportion of impurities in the incoming gas, the faster a stable periodic process comes in isothermal and thermocontrolled modes: impurities accumulate faster to a steady value. In general, about 20 cycles in all considered modes are sufficient to achieve an approximately steady value of the combustion energy, however, the accumulation of ethane in the considered processes continues after 20 cycles.

These results are adequately consistent with the publications reviewed in the introduction. In most studies, the noticeable effect of ethane, which is the main impurity of natural gas, ended approximately in the region of 10–30 cycles [6,7,10]. In terms of the effect on the active capacitance, the results are also close to those published earlier: in [7], the effect of 10.3% mol. impurities (not only ethane) led to a loss of 20% capacity, and in [10] the effect of 10.4%mol. impurities to losses of 16%, which correlates with the simulation results. In general, such an approach can be considered justified, at least as a first approximation.

**Figure 16:**
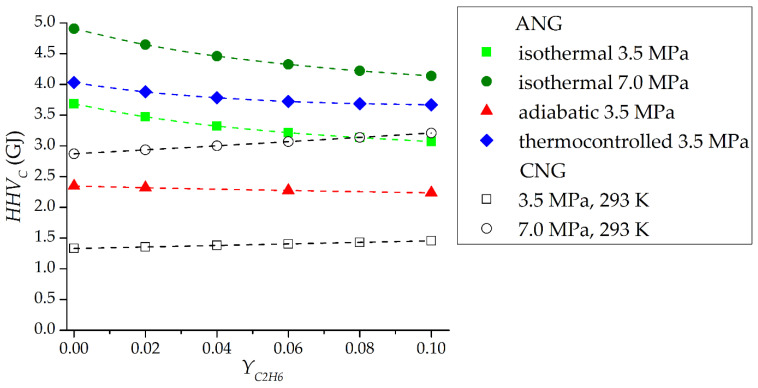
Dependence of the active energy capacity of the gas leaving the storage with a volume of 1 m^3^ for 1 cycle based on ACPK adsorbent based on the results of modeling cyclic processes. ANG is the adsorption gas storage. CNG is the conventional gas storage in the “isothermal” mode, without taking into account non-equilibrium intake/exhaust processes.

Figure 16 shows the dependencies of the steady-state values of the active energy capacity of the adsorption and conventional gas storage for one charging and discharging cycle on the ethane content in the supplied gas for different modes. As the ethane content increases, the energy capacity of the gas storage increases by increasing the density of the mixture–or by increasing the average molar energy of combustion. The efficiency of the adsorption storage, on the contrary, decreases. It is likely that there is a certain concentration of ethane at which adsorption storage will no longer provide an advantage over conventional gas storage. In Figure 16, this is clearly seen at a pressure of 7.0 MPa: with an ethane fraction of 10% mol. adsorption storage, which gives only a small advantage in active energy capacity compared to conventional gas storage: 29% in isothermal mode, although with pure methane the advantage is 71%. At a pressure of 3.5 MPa, the advantage of the adsorption storage is more obvious: even in the low-efficiency adiabatic mode, the energy capacity is higher than that of the conventional gas storage, and in the thermocontrolled mode, the active energy capacity is 2.5 times greater at an ethane concentration in the gas of 10% mol. and three times greater when operating on pure methane.

The simulation results show that the area of effective application of adsorption storages on real natural gas with impurities is smaller than in the case of analyzing their operation on pure methane. Ignoring impurities during the design phase of the adsorption storage can lead to high and unrealistic expectations and will negatively affect real work. It should be noted that the dealing with impurities is possible not only due to the thermal management analyzed in the article, but also using various scheme solutions of the adsorption storage tank.

## 4. Conclusions

Synthesis of active carbon ACPK from peat raw materials was carried out. The resulting adsorbent has a wide pore size distribution with maxima in the region of a pore radius of 0.59 (main extremum) and 0.92 nm (secondary). The specific volume of micropores is 0.44 cm^3^/g with a total pore volume of 0.56 cm^3^/g. The adsorption of methane at 3.5 MPa and 293 K was 10.5% wt., which makes it possible to use this adsorbent for the purpose of methane storage under the condition of a high packing density of the adsorbent–for example, in the form of monoliths. Theoretical studies of a binary mixture of methane–ethane (with an ethane content of up to 10 mol.) adsorption were carried out by the IAST method based on experimental adsorption isotherms of pure components. It is shown that at a pressure of 3.5 MPa and 293 K, the higher heating value of the accumulated adsorbate at an ethane concentration of 10% mol. in the gas phase is 8.57 MJ per 1 kg of adsorbent, while in the case of pure methane this heat is 5.84 MJ/kg, 1.47 times less. Thus, capturing ethane (and other heavy hydrocarbons), for example, from a flowing natural gas stream can significantly increase the capacity of the adsorption storage, provided that the accumulated gases can later be recovered.

Numerical molecular modeling of the methane–ethane mixture adsorption in slit-like model micropores of various widths from 0.6 to 1.8 nm at a temperature of 293 K was carried out. It is shown that this mixture is characterized by the appearing characteristic molecular effects: the displacement of ethane by methane molecules from narrow 1.0 nm-wide micropores, as well as the formation of obvious layers of methane and ethane in wide micropores 1.8 nm wide. However, the integral molecular adsorption isotherm of the mixture, which includes adsorption in pores of various sizes, corresponding to the result of the QSDFT method, is similar in shape to the adsorption isotherm, determined by the IAST method. Thus, various molecular effects observed in individual model micropores together result in the classical adsorption isotherm.

Since the results of numerical molecular modeling showed no contradictions with the IAST model, this approach was used to simulate the cyclic processes of gas charging and discharging from a 1 m^3^ adsorption storage filled with ACPK adsorbent. Three modes of operation were analyzed: adiabatic, isothermal, and thermocontrolled, which is the opposite of adiabatic. The results of the study showed that in the adiabatic mode, the accumulation of ethane can continue up to about 110 cycles, although the accumulation itself had very little effect on the higher heating value of the gas discharging in the cycle. To a much greater extent, the thermal effects of adsorption themselves were influential, reducing the energy capacity by 27–33%. The thermocontrolled process showed significant efficiency compared to the isothermal one: if on pure methane the advantage in energy capacity was 9.4%, then at 10% mol. ethane the share advantage increased by 19.5% due to a more efficient ethane recovery. Thus, thermal management (heating during discharging) of the adsorption storage is a viable way not only to directly increase the amount of outgoing gas, but also an effective means of dealing with the accumulation of ethane. The analysis of the active energy capacity of the adsorption storage in comparison with a conventional gas one showed that at a relatively high pressure of 7.0 MPa, the capacity of the adsorption storage is 71% higher in the isothermal mode on pure methane and only 29% higher when operating on natural gas with a content of 10% mol. ethane. Thus, it is not enough to evaluate the efficiency of the adsorption storage solely for pure methane; it is necessary to take into account the actual composition of the gas. At a pressure of 3.5 MPa, the efficiency of the adsorption storage is significantly higher than that of the conventional gas storage, especially in the thermocontrolled mode: the active energy capacity is 2.5 times higher at an ethane concentration in the gas of 10% mol. and three times higher when operating on pure methane.

## Figures and Tables

**Table 1:** Parameters of the microporous structure of the ACPK carbon adsorbent calculated from SAXS data for various pore models.

Pore Model	Spherical		Cylindrical	
Model characteristics	*R_G_*	*R*_S_, nm	*R_G_*	*R_T_*, nm
ACPK	0.40	0.52	0.22	0.31

**Table 2:** Elemental chemical composition of the ACPK surface.

Content	C	O	Other Impurities
% at.	75.6	18.3	6.1
% wt.	65.6	21.1	13.3

**Table 3:** Parameters of the porous structure of the carbon nanoporous adsorbent ACPK.

Method	BET	TVFM	TVFM	TVFM	TVFM	TVFM	BET	QSDFT
Adsorbent	*S*_BET_, m^2^/g	*W*_0_, cm^3^/g	*x*_0_, nm	*E*_0(N2)_, kJ/mol	*W*_S_, cm^3^/g	*W*_ME_, cm^3^/g	*S*_ME_, m^2^/g	*r_max_*, nm
ACPK	1105	0.44	0.52	7.63	0.56	0.12	40	0.59

## Data Availability

Not applicable.
